# Who could complete and benefit from the adjuvant chemotherapy regarding pancreatic ductal adenocarcinoma? A multivariate‐adjusted analysis at the pre‐adjuvant chemotherapy timing

**DOI:** 10.1002/cam4.4698

**Published:** 2022-04-17

**Authors:** Ningzhen Fu, Kai Qin, Jingfeng Li, Jiabin Jin, Yu Jiang, Xiaxing Deng, Baiyong Shen

**Affiliations:** ^1^ Pancreatic Disease Center, Department of General Surgery Ruijin Hospital, Shanghai Jiaotong University School of Medicine Shanghai China; ^2^ Research Institute of Pancreatic Disease Shanghai Jiaotong University School of Medicine Shanghai China; ^3^ State Key Laboratory of Oncogenes and Related Genes Shanghai China; ^4^ Institute of Translational Medicine Shanghai Jiaotong University Shanghai China

**Keywords:** chemotherapy completeness (CHC), fasting blood glucose, FBG, pancreatic ductal adenocarcinoma (PDAC), pre‐adjuvant chemotherapy (PAC), sarcopenia

## Abstract

**Background:**

The pre‐adjuvant chemotherapy (PAC) status of postoperative pancreatic ductal adenocarcinoma (PDAC) patients has not been studied and elaborated well previously.

**Method:**

The association of PAC variables and prognoses was explored using a multivariable Cox model, restricted cubic spline analysis, and correlation analysis. The main outcomes were overall survival (OS) and progression‐free survival (PFS). The secondary outcome was chemotherapy completeness (CHC).

**Results:**

A total of 401 eligible patients were enrolled in sequential surgery and chemotherapy. The chemotherapy regimen, PAC fasting blood glucose (FBG), and elevated fasting blood glucose (eFBG) status were associated with CHC (regimen types: *p* = 0.005, continuous FBG: *p* = 0.014, eFBG status: *p* = 0.012). Early administration of adjuvant chemotherapy (<34 days) was a risk factor for the limited OS and PFS (OS: aHR: 1.61 [1.09–2.38], *p* = 0.016; PFS: aHR: 1.91 [1.29–2.82], *p* = 0.001). Patients with higher PAC body mass index (BMI), receiving Gemcap regimen, and with lower PAC tumor marker value were observed with better survival prognoses (PAC BMI: OS: 0.927 [0.875–0.983], *p* = 0.011; Gemcap: OS: 0.533 [0.312–0.913], *p* = 0.022; Gemcap: PFS: 0.560 [0.341–0.922], *p* = 0.023; PAC CA125: OS: 1.004 [1.002–1.006], *p* < 0.001; PAC CA125: PFS: 1.003 [1.000–1.005], *p* = 0.031; PAC CEA: OS: 1.050 [1.026–1.074], *p* < 0.001). The BMI decrease was mainly concentrated in the first 3 months of chemotherapy courses (first 3 months: *p* < 0.001; latter 3 months: *p* = 0.097). And CEA, compared to CA125 and CA199, was a better prognostic indicator (CEA: first 3 months: PFS *p* = 0.011, OS *p* < 0.001; latter 3 months: PFS *p* = 0.024, OS *p* = 0.041).

**Conclusion:**

PDAC patients should be treated with adjuvant chemotherapy over 34 postoperative days. PAC sarcopenia was a risk factor for OS, but not PFS and limited CHC. Those with higher PAC FBG levels were more likely to finish chemotherapy. CEA, compared to CA125 and CA199, was a better prognostic indicator.

## Graphical abstract

The pre adjuvant chemotherapy status (PACS) was important in determining the chemotherapy completeness and the chemotherapy benefits. However, no previous studies were published or in process regarding this issue. In this study, based on the multivariate adjusted analysis, the restricted cubic spline curve, and the correlation analysis, we found the unfavorable roles of PACS sarcopenia and early‐chemotherapy‐administration for survival prognoses. In addition, it was discovered that patients with high PACS fasting blood glucose were more likely to finish more chemotherapy courses.

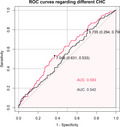

## 1 BACKGROUND

Pancreatic cancer (PC), one of the most aggressive and lethal malignancies, is characterized by high mortality, frequent recurrence, and limited survival.^1^ Less than 9% of PC patients could survive 5 years after diagnosis.^1^ The major histological subtype of PC is pancreatic ductal adenocarcinoma (PDAC), which accounts for approximately 85% of cases.^2^ Surgery is the only option to cure the disease, and adjuvant chemotherapy is strongly recommended.^3^, ^4^ However, most patients could not complete the chemotherapy courses, and many of them experienced tumor recurrence within half a year.^5^, ^6^

Surgical trauma during pancreatic surgery is heavy,^7^, ^8^ and complications of pancreatic surgery are frequent.^9^, ^10^, ^11^, ^12^, ^13^ Thus, the recovery periods of patients with PDAC varied significantly.^7^, ^8^ Further, the timing of chemotherapy administration differs among these patients. Though it is ever reported that time to starting chemotherapy did not influence overall survival rates,^14^ it is not studied and discussed in the East Asian population. Despite this, pre‐adjuvant chemotherapy (PAC) timing could be appropriate for evaluating chemotherapy tolerance and survival prognosis. Similar studies regarding the preoperative timing were processed.^15^, ^16^, ^17^, ^18^, ^19^ Nevertheless, no relevant studies regarding PAC timing have been published or in process until now. It is still uncertain whether the status of patients at this stage affected the patients' prognoses. In this study, we focused on patients' PAC status to explore their role in PDAC patient prognoses.

## 2 METHOD

### 2.1 Data collection

All eligible patients diagnosed as PDAC were consecutively enrolled from the Pancreatic Disease Center, Ruijin Hospital Affiliated to Shanghai Jiao Tong University School of Medicine between January 2013 and December 2019. The inclusive criteria included: (1) pathologically diagnosed as PDAC; (2) resectable PDAC according to NCCN guideline^3^; (3) aged between 18 and 85 years old; (4) receiving radical pancreatectomy surgery; (5) receiving adjuvant chemotherapy. The exclusion criteria were as follows: (1) absence of body mass index (BMI) data at diagnosis; (2) incomplete oncological data (including tumor size, examined lymph node, positive lymph node, differentiation); (3) without regular follow‐up; (4) heterogenous carcinoma; (5) no history of adjuvant chemotherapy in the Ruijin Hospital; (6) administered with neoadjuvant therapy. Finally, 401 patients were enrolled in our study (Figure S1).

The study protocol was approved by the Institutional Review Board of the authors' affiliated hospital. The local ethics committee waived the need for informed consent because the study was observational and retrospective. The study was conducted according to the strengthening the reporting of observational studies in epidemiology (STROBE) guidelines^20^ and in accordance with the latest version of the Declaration of Helsinki.

### 2.2 Definition

Height and weight information were recorded during the diagnosis. BMI was calculated as weight in kilograms divided by the square of height in meters. Sarcopenia was defined as BMI below 18.5 kg/m^2^.^21^ The disease was staged according to the 8th American Joint Committee on Cancer using the TNM system. The regimens of adjuvant chemotherapy were subdivided into gemcitabine + capecitabine (Gemcap),^3^, ^22^ gemcitabine + S‐1 (GS),^23^, ^24^, ^25^, ^26^, ^27^ gemcitabine alone (Gem),^3^ and other groups. The other group included albumin paclitaxel + gemcitabine, mFolfirinox, capecitabine + oxaliplatin, and gemcitabine + oxaliplatin. Patients were followed up with detailed variables recorded at *the beginning* (before the first chemotherapy course started), *mid‐term* (3 months since the first course), and *end* (when completing the 6‐month chemotherapy administration). PAC timing referred to the same day but before administrated with the first adjuvant chemotherapy cycle. After *the end* of follow‐up, regular follow‐up regarding recurrence and survival status was regularly performed every 3 months. Chemotherapy completeness (CHC) was categorized as less than half (LF) (quit chemotherapy or change regimens before *mid‐term* follow‐up), more‐than‐half (MF) (quit chemotherapy or change regimens after *mid‐term* but before *end* follow‐up), and completed (CP) (complete adjuvant chemotherapy courses with the initial regimen). Recurrence or intolerance was recorded per adjacent radiological examination of the nearest chemotherapy course or a shift in chemotherapy regimens (Table S1). Elevated fasting blood glucose (eFBG) level was defined as over 7 mmol/L at PAC.^28^ Pre‐chemotherapy period (PCP) was defined as the time from surgery to the first administration of chemotherapy. Categorized PCPs were further grouped based on the X‐tile PCP cutoff (34 days). Overall survival (OS) in months was defined as the time from diagnosis to the date of death due to any cause. Progression‐free survival (PFS) in months was defined as the time from diagnosis to the date of radiological progression observation.

### 2.3 Statistical analysis

The primary outcomes were OS and PFS. The secondary outcome was CHC. Despite of PCP and PAC variables, the change of variables during adjuvant chemotherapy courses was also studied and analyzed.

Normally distributed continuous variables are presented as mean ± SD and analyzed using Student's *t*‐test. Non‐normally distributed continuous variables were presented as median (Q1–Q3) and analyzed using the Mann–Whitney *U* test. The Kolmogorov–Smirnov test was used for the normality tests of continuous variables (Table S2). Receiver operator characteristic (ROC) curve and the area under the curve (AUC) were utilized for further evaluation of the association between FBG and CHC. Categorical variables are presented as percentages and analyzed using Pearson's test. The Wilcoxon sum rank test was used for pairwise comparisons between non‐normally distributed factors. OS and PFS were assessed using Kaplan–Meier curves and log‐rank tests in univariable analyses and restricted cubic spline (RCS) curves^29^, ^30^, ^31^ and Cox proportional hazards models in multivariable analyses adjusted for clinically relevant factors identified in the univariable analyses. The hazard ratio (HR) and adjusted hazard ratio (aHR) are displayed with a 95% confidence interval (CI). The Cox–Mantel test was used for significance comparison. The knot number for the RCS curves was set to 5 because of the sample size. The X‐tile was used to determine the cutoff value.^32^ Association between categorical data was assessed using the *χ* ^2^ test, while association between continuous data was assessed using the Spearman rank test, and the association between categorical data and continuous data was assessed using the Kruskal–Wallis test.

Statistical analysis was performed using SPSS (IBM SPSS Statistics 26.0), R Studio, and X‐Tile software. *p* < 0.050 was regarded statistically significant.

## 3 RESULTS

**FIGURE 1:**
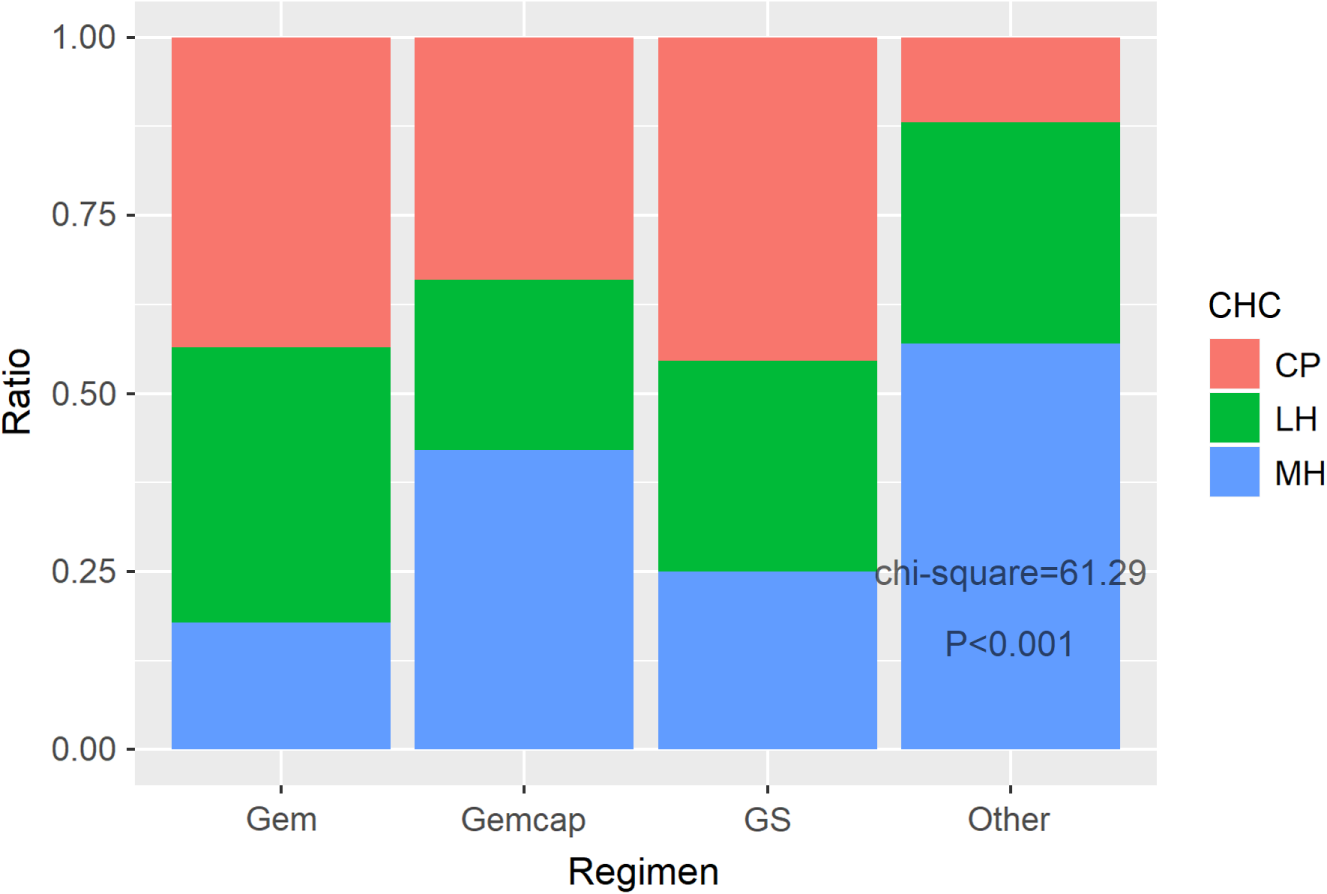
The ratio of different CHC regarding different chemotherapy regimens

**FIGURE 2:**
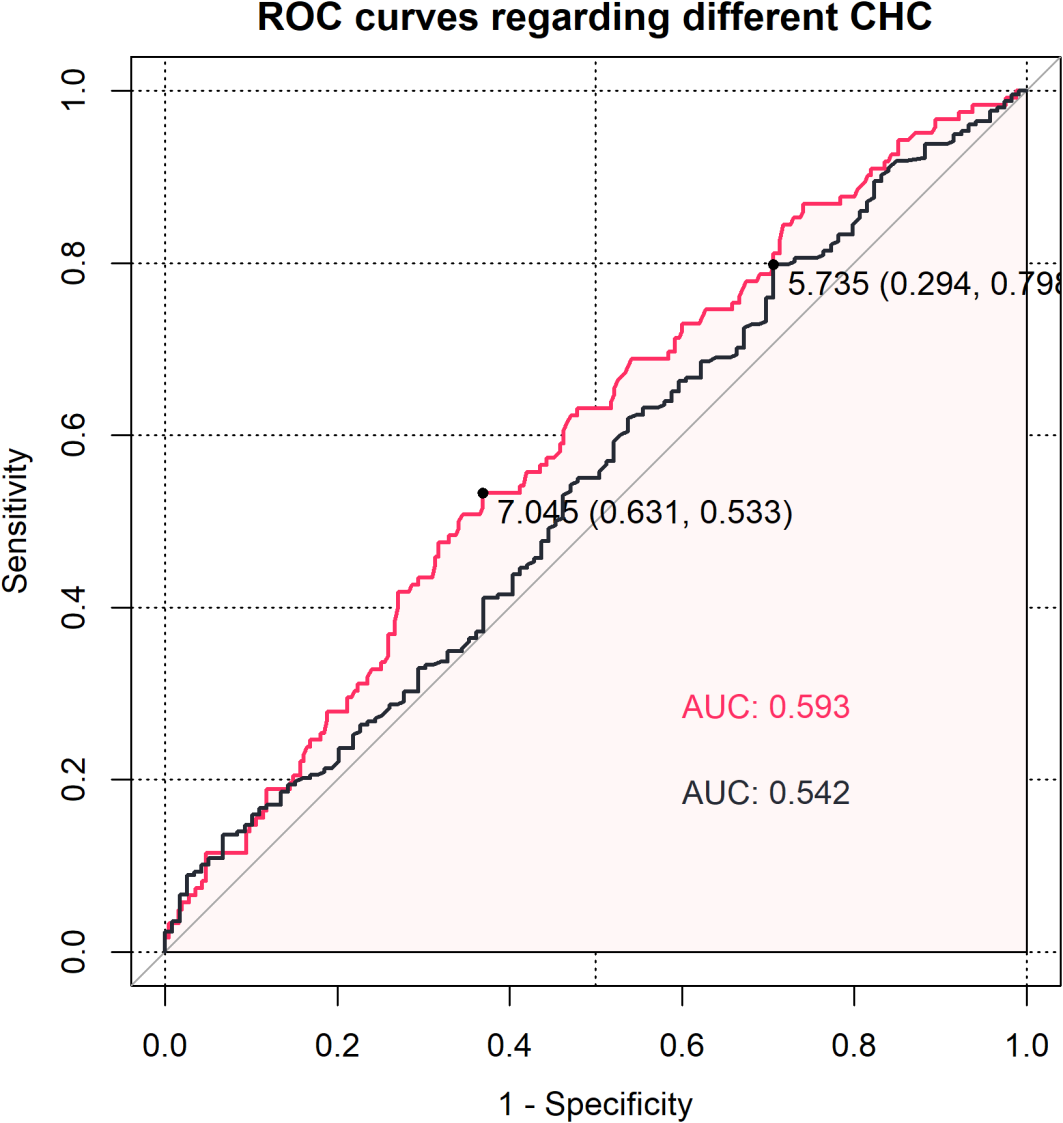
The ROC curves of PAC FBG regarding CP versus MH + LH (red) and MH + CP versus LH (black) CHC

A total of 401 eligible patients were included in this study (Figure S1), and their baseline data are displayed in Table 1. Only 31.7% patients finished the whole adjuvant chemotherapy courses and 68.3% finished over a half. The patients with eFBG in different CHC groups were 48 (37.8%, LH), 48 (32.7%, MH), and 65 (51.2%, CP), respectively. The CHC was found to be closely related to regimen types, PAC fasting blood glucose (FBG), and eFBG status at PAC (regimen types: *p* = 0.005 [Figure 1], PAC FBG: *p* = 0.014 [Figure 2], eFBG status: *p* = 0.012). Nevertheless, the BMI, albumin (Alb), and prealbumin (PreAlb) at PAC were not found to be relevant with CHC (BMI: *p* = 0.079, Alb: *p* = 0.300, PreAlb: *p* = 0.507). Moreover, among the BMI, Alb, and PreAlb, significant associations were observed (BMI‐Alb *p* = 0.017, BMI‐PreAlb *p* = 0.003, and Alb‐PreAlb *p* < 0.001). Along with the chemotherapy process, the BMI, body weight (BW), Alb, CA199, and CA125 decreased, while CEA increased (Table S3).

**TABLE 1:** Baseline data at PAC, 3‐month follow‐up, and the last cycle

	PAC (*n* = 401) (100%)	3‐month follow‐up (*n* = 274) (68.3%)	Last cycle (*n* = 127) (31.7%)
Age (years)	61 (56–65)	61 (56–65)	61 (57–65)
Female (%)	143 (35.7)	91 (33.2)	41 (32.3)
BMI (kg/m^2^)	20.55 (2.36)	20.25 (2.46)	20.37 (2.53)
CA199 (U/ml)	15.85 (6.50–37.60)	12.6 (5.57–31.80)	11.00 (5.05–29.20)
CA125 (U/ml)	26.40 (15.75–49.52)	13.00 (8.50–19.85)	11.50 (8.30–16.10)
CEA (ng/ml)	2.16 (1.50–3.33)	2.95 (1.83–4.93)	2.93 (1.81–4.75)
Alb (g/L)	41 (38–43)	39 (36–42)	39 (37–42)
PreAlb (mg/L)	198 (162–226)	202 (164–240)	200 (164–238)
FBG (mmol/L)	6.69 (5.78–8.58)	6.80 (5.58–8.77)	7.29 (5.79–8.91)
PCP (d)	50 (40–62)	50.5 (41–63)	50 (42–62)
Regimen			
Gem	101 (25.2)	62 (22.6)	44 (34.6)
GS	108 (26.9)	76 (27.7)	49 (38.6)
Gemcap	50 (12.5)	38 (13.9)	17 (13.4)
Other	142 (35.4)	98 (35.8)	17 (13.4)
Stage			
I	62 (15.5)	43 (15.7)	6 (4.7)
II	305 (76.1)	211 (77)	118 (92.9)
III	34 (8.5)	20 (7.3)	3 (2.4)
Differentiation			
Poor	254 (63.3)	173 (63.1)	80 (63.0)
Moderate	146 (36.4)	101 (36.9)	47 (37.0)
Well	1 (0.2)	0 (0)	0 (0)

Abbreviations: Alb, albumin; BMI, body mass index; FBG, fasting blood glucose; Gem, gemcitabine only; Gemcap, gemcitabine + capecitabine; GS, gemcitabine + S‐1; PAC, pre‐adjuvant chemotherapy; PreAlb, prealbumin.

**FIGURE 3:**
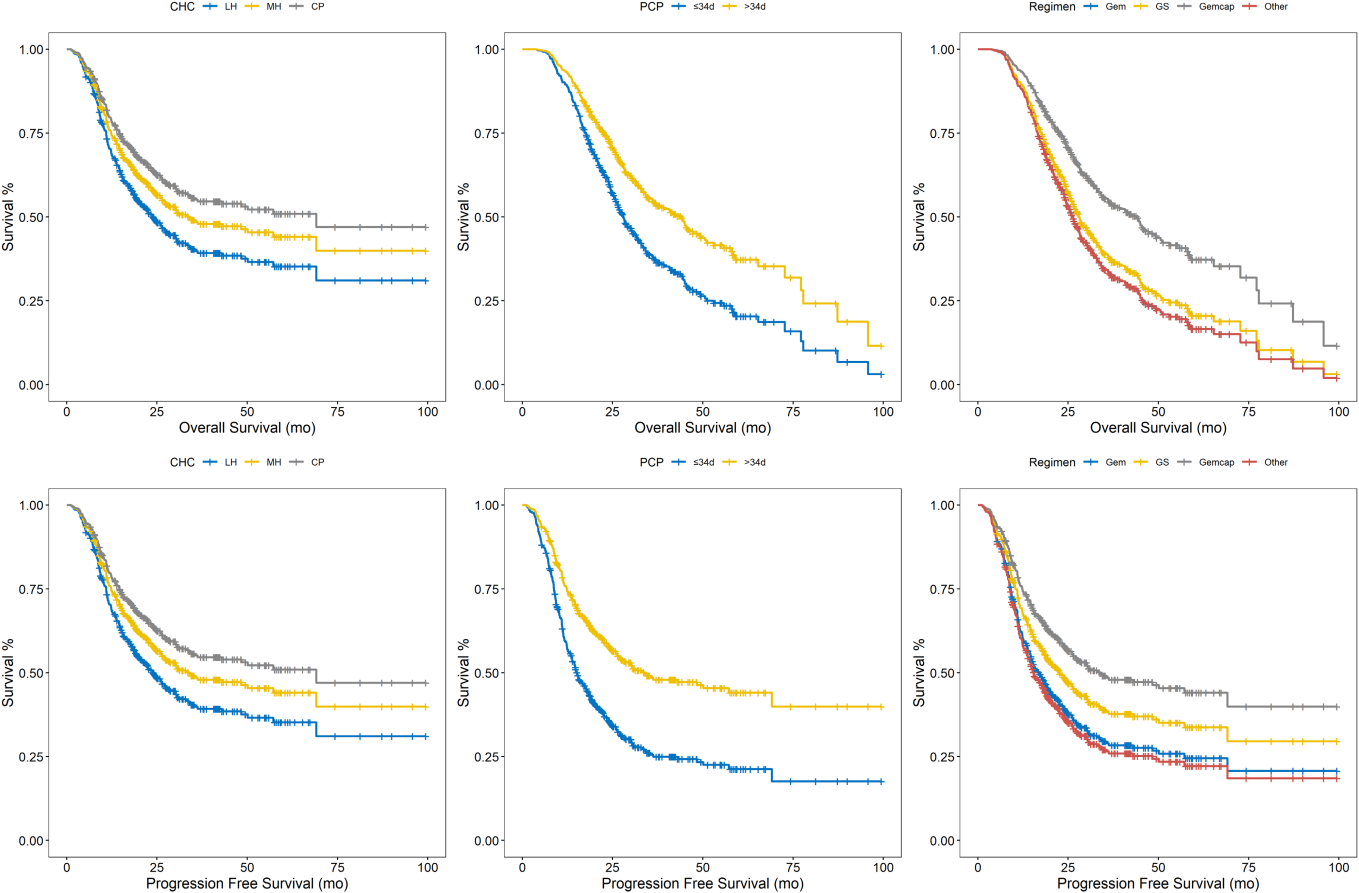
The adjusted survivorship curves of Cox model with CHC, categorical PCP, and regimens regarding OS and PFS, respectively

**FIGURE 4:**
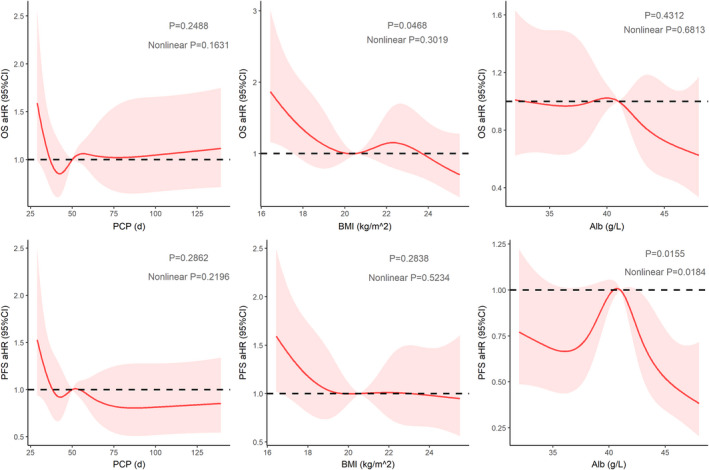
The RCS curves of PCP, PAC BMI, and PAC Alb regarding OS and PFS, respectively

In univariate Cox analyses, PAC BMI, PAC CA199, PAC CA125, PAC CEA, PAC Alb, regimen types, TNM stage, CHC, and categorized PCP were significantly associated with both OS and PFS, while differentiation was only significantly associated with OS (Table 2). Further, in multivariate Cox model regarding OS, PAC BMI, PAC CA125, PAC CEA, regimen type, stage, differentiation, CHC, and categorized PCP maintained their prognostic impact on OS (Table 2; Figure 3). In PFS multivariate Cox model, PAC CA199, PAC CA125, stage, categorical PCP, and CHC manifested impact significance (Table 2; Figure 2). Continuous PCP, however, was not relevant with CHC (*p* = 0.480) and survival prognoses. Regarding the PAC BMI, PAC Alb, and PCP, RCS was performed to evaluate the impact of these variables on OS and PFS at different levels (Figure 4).

**TABLE 2:** Univariate and multivariate Cox analyses regarding OS and PFS

	Univariate OS		Multivariate OS		Univariate PFS		Multivariate PFS	
	HR	*p*	aHR	*p*	HR	*p*	aHR	*p*
Age	1.008 (0.992–1.025)	0.321			1.004 (0.988–1.020)	0.605		
Female	0.974 (0.748–1.267)	0.843			0.983 (0.863–1.119)	0.797		
PAC BMI	0.916 (0.867–0.967)	0.002	0.927 (0.875–0.983)	0.011	0.946 (0.897–0.998)	0.042	0.954 (0.903–1.009)	0.101
PAC CA199	1.000 (1.000–1.000)	0.019	1.000 (1.000–1.000)	0.162	1.000 (1.000–1.000)	<0.001	1.000 (1.000–1.000)	0.053
PAC CA125	1.006 (1.004–1.008)	<0.001	1.004 (1.002–1.006)	<0.001	1.004 (1.002–1.006)	<0.001	1.003 (1.000–1.005)	0.031
PAC CEA	1.045 (1.033–1.058)	<0.001	1.050 (1.026–1.074)	<0.001	1.030 (1.016–1.043)	<0.001	1.008 (0.988–1.028)	0.448
PAC Alb	0.960 (0.931–0.990)	0.009	0.975 (0.944–1.007)	0.124	0.963 (0.935–0.992)	0.012	0.971 (0.942–1.001)	0.059
PAC PreAlb	1.000 (0.997–1.003)	0.901			1.000 (0.997–1.003)	0.896		
PAC FBG	0.992 (0.949–1.037)	0.716			0.991 (0.949–1.034)	0.667		
PCP	1.001 (0.998–1.004)	0.502			0.998 (0.994–1.002)	0.411		
PCP > 34 d	0.649 (0.446–0.947)	0.025	0.620 (0.420–0.915)	0.016	0.523 (0.364–0.751)	<0.001	0.524 (0.355–0.774)	0.001
Regimen		0.068		0.107		0.027		0.039
Gem	Ref		Ref		Ref		Ref	
GS	0.986 (0.690–1.409)	0.938	0.874 (0.601–1.270)	0.479	0.835 (0.591–1.180)	0.307	0.768 (0.537–1098)	0.148
Gemcap	0.547 (0.332–0.904)	0.018	0.533 (0.312–0.913)	0.022	0.559 (0.348–0.897)	0.016	0.560 (0.341–0.922)	0.023
Others	1.044 (0.767–1.419)	0.785	1.000 (0.682–1.467)	0.999	1.077 (0.793–1.462)	0.635	1.063 (0.730–1.550)	0.749
Stage		<0.001		0.006		0.001		0.003
I	Ref		Ref		Ref		Ref	
II	1.042 (0.740–1.466)	0.816	1.197 (0.793–1.806)	0.392	0.955 (0.680–1.341)	0.791	1.247 (0.828–1.877)	0.291
III	2.606 (1.619–4.194)	<0.001	2.192 (1.307–3.678)	0.003	2.130 (1.305–3.479)	0.003	2.419 (1.410–4.149)	0.001
Differentiation		0.056		0.089		0.646		
Poor	Ref		Ref		Ref			
Moderate	0.738 (0.564–0.966)	0.027	0.741 (0.555–0.988)	0.041	0.885 (0.684–1.144)	0.952		
Well	2.342 (0.327–16.783)	0.397	2.175 (0.295–16.029)	0.446	0 (0–7.469E+138)	0.351		
CHC		<0.001		0.001		0.001		0.026
LH	Ref		Ref		Ref		Ref	
MH	0.605 (0.455–0.804)	0.001	0.721 (0.526–0.987)	0.041	0.746 (0.558–0.997)	0.048	0.787 (0.575–1.076)	0.133
CP	0.410 (0.295–0.568)	<0.001	0.509 (0.359–0.722)	<0.001	0.559 (0.408–0.765)	<0.001	0.639 (0.460–0.888)	0.008

Abbreviations: OS, overall survival; PFS, progression‐free survival; HR, hazard ratio; aHR, adjusted hazard ratio; PAC, pre‐adjuvant chemotherapy; BMI, body mass index; Alb, albumin; PreAlb, prealbumin; FBG, fasting blood glucose; Gem, gemcitabine only; GS, gemcitabine + S‐1; Gemcap, gemcitabine + capecitabine; CHC, chemotherapy completeness; LH, less‐than‐half; MH, more‐than‐half; CP, completed.

**FIGURE 5:**
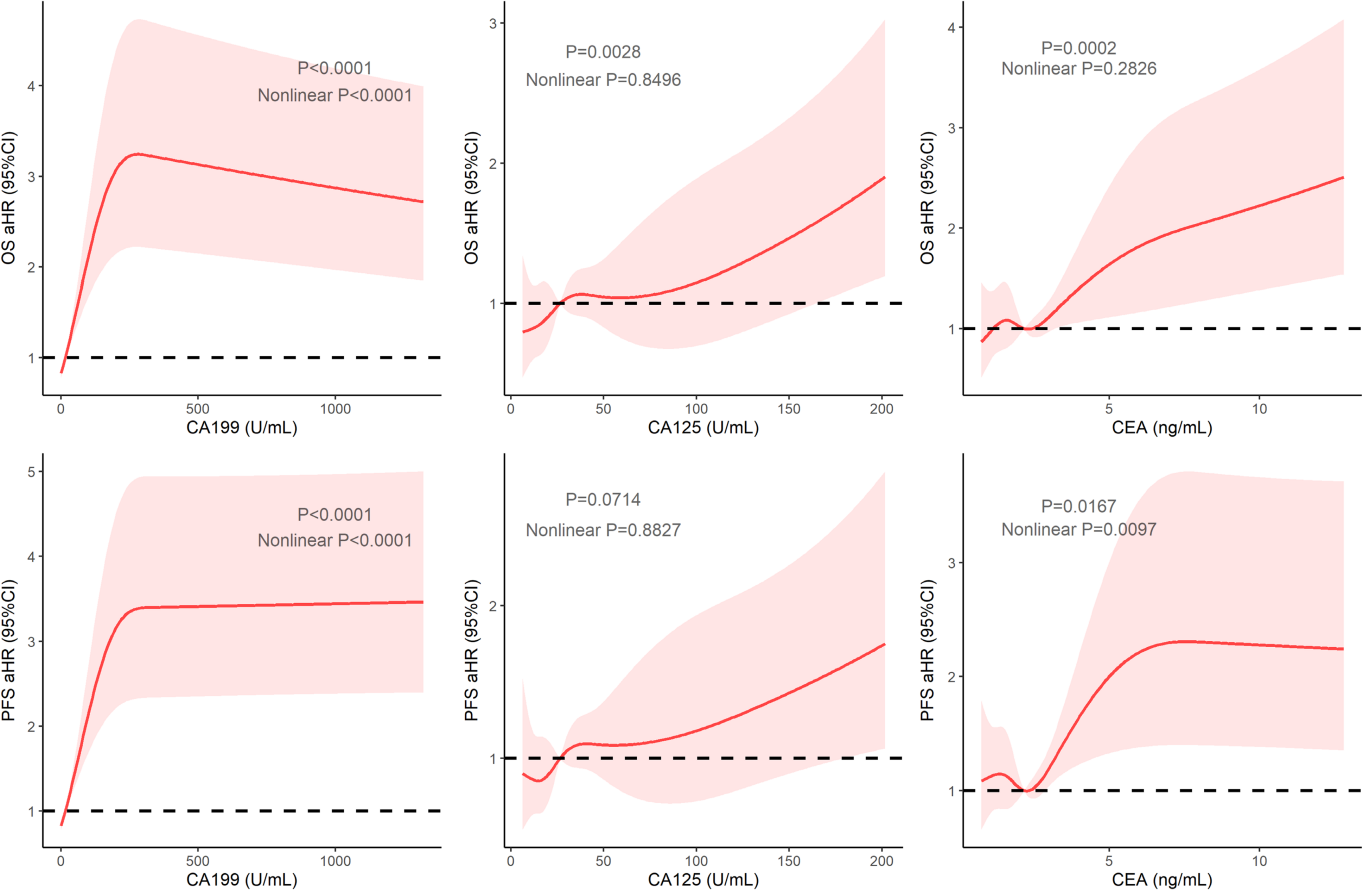
The RCS curves of PAC CA199, PAC CA125, and PAC CEA regarding OS and PFS, respectively

Tumor markers were vital variables for monitoring the progression of PC in follow‐ups. At PAC, CA125, and CEA levels were risk factors for OS. Only CA125 was a risk factor for PFS (Table 2). Then, we explored the roles of the changes of these tumor markers. The decrease in CEA indicated better OS and PFS survival in the multivariate Cox models (Table 3). The RCS of the PAC tumor markers are displayed in Figure 5.

**TABLE 3:** Multivariate Cox models integrated with tumor marker decreases. (Other variables were concealed)

	Decrease during first 3 months				Decrease during last 3 months			
	OS		PFS		OS		PFS	
	aHR	*p*	aHR	*p*	aHR	*p*	aHR	*p*
CA199	1.000 (1.000–1.000)	0.711	1.000 (1.000–1.000)	0.113	0.996 (0.994–0.999)	0.003	0.999 (0.995–1.002)	0.365
CA125	0.995 (0.990–1.000)	0.066	0.992 (0.970–1.015)	0.496	0.985 (0.923–1.050)	0.643	1.009 (0.958–1.064)	0.727
CEA	0.962 (0.944–0.981)	<0.001	0.994 (0.989–0.999)	0.011	0.845 (0.749–0.954)	0.007	0.887 (0.799–0.984)	0.024

Abbreviations: aHR, adjusted hazard ratio; OS, overall survival; PFS, progression‐free survival.

## 4 DISCUSSION

All PDAC patients undergoing radical surgeries should be treated with adjuvant chemotherapy, which is vital to cure the disease and prolong survival. Gemcap is the guideline‐recommended regimen,^3^, ^22^ and GS is an alternative regimen designed for Asian patients.^23^, ^24^, ^25^, ^26^, ^27^ As shown in Figure 1, the CHC was higher in the Gem and GS groups, which represented better chemotherapy tolerance. Gemcap manifested the best performance in prolonging OS and PFS, which corresponded with the ESPAC‐4 study.^6^, ^22^ No significant differences between GS and Gem alone were observed, which was consistent with the GEST study.^23^

The CHC was discovered to be essential for patient survival prognoses in the study. However, among patients receiving adjuvant chemotherapy, only approximately 1/3 of them could finish the course. Altman et al. reported a similar phenomenon in that completion of chemotherapy was associated with improved OS, and only 7% of patients could complete that.^33^ We sought to determine whether CHC could be predicted. Despite the regimen type, PAC FBG and PAC eFBG levels were significantly associated with CHC. Zarei et al. proposed that hypoglycemia could induce chemoresistance via HuR‐IDH1‐mediated redox balance in vitro and in vivo.^34^ Further, they discovered the improved survival of patients receiving adjuvant gemcitabine with elevated serum glucose levels.^34^ However, the impact of elevated FBG levels on CHC has not yet been discussed. In our study, although not significantly associated with improved survival, higher FBG and the eFBG status indicated more complete CHC, which corresponded with the mechanism proposed by Dr. Zarei. As single variable model, the AUC of FBG was acceptable but not satisfactory enough. The model was potential to be modified and enhanced with more related variables integrated in the future. The RCS curves of PAC FBG regarding OS, PFS, and CHC (CP) was plotted in Figure S2. Among the range of 0–16 mmol/L FBG, it appears that the higher the PAC FBG was, the better the CHC could be. The inflection point is around 10 mmol/L. Nevertheless, the PAC BMI, PAC Alb, and PAC PreAlb, which were associated with patients' physical status, manifested no relevance to CHC. Notably, the associations among the three variables were strongly significant.

Along with the processing of chemotherapy, BMI, Alb, and PreAlb continued to decrease. The significant decrease mainly occurred during the first 3 months, suggesting that the maintenance of body weight and physical status should be especially emphasized in the first 3 months. Stratified by CHC, for the three variables, the phenomena remained the same in the CP and MH subgroups (all *p* < 0.001). Hashimoto et al. discovered that the body weight loss happened mostly during the first two postoperative months, which was consistent with our results.^35^ They also proposed that severe body weight loss was associated with survival prognoses, whereas no association was found in our study (*p* = 0.390).^35^ In their study, they categorized body weight loss and did not analyze the continuous body weight loss factor. In addition, adjustment of covariables or propensity score balance was not applied in the analyses. This could account for the divergence between our results. Morita et al. reported that body weight loss could affect chemotherapy continuity,^36^ which differed from our results (BMI change‐CHC association *p* = 0.323). They evaluated the chemotherapy continuity with the relative dose intensity (RDI) factor indirectly and categorized body weight loss with a cutoff of 10%.^36^ Moreover, the association between RDI and categorized body weight loss in their study was 0.039, and the sample size of the study was limited (only 54 cases).^36^

In univariate Cox analyses, BMI, Alb, and categorical PCP were found to be relevant to both OS and PFS. In multivariate models, high BMI was a protective factor for OS, while early administration of chemotherapy (PCP ≤ 34 days) was a risk factor in both OS and PFS models. RCS analyses were processed with respect to BMI, Alb, CA199, CA125, CEA at PAC, and PCP. In PCP RCS plots, the risk decreased initially and reached the bottom during the 30–40‐day period. The curve then rebounded slightly and remained horizontal. The 95% CI area included the aHR = 1 auxiliary line, indicating that no significant association existed between continuous PCP values and OS/PFS. In another word, it does not matter a lot for patients to be administrated with adjuvant chemotherapy at what time after 34 postoperative days. Similarly, PAC BMI RCS curves indicated that sarcopenia was a risk factor for OS, which corresponded with the discovery of Dr. Rom.^16^ Unlike Dr. Rom, who chose the preoperative timing, the timing of PAC was studied in this study. Unexpectedly, PAC Alb at approximately 40 g/L might be unfavorable for both OS and PFS (Alb OS: total *p* = 0.4312 nonlinear *p* = 0.6813; Alb PFS: total *p* = 0.0155, nonlinear *p* = 0.0184). As for tumor markers, CA199, with no doubt, was still the most influential and would reach the plateau at around 200–300 U/ml. As for CA125 and CEA, along with an increase in their values, the risk increased smoothly and linearly, except for CEA, regarding PFS (CA125 OS: total *p* = 0.0028, nonlinear *p* = 0.8496; CA125 PFS: total *p* = 0.0714, nonlinear *p* = 0.8827; CEA OS: total *p* = 0.0002, nonlinear *p* = 0.2826; CEA PFS: total *p* = 0.0167, nonlinear *p* = 0.0097).

The association between changes in tumor markers and prognosis was further explored. CA199, CA125, and CEA are the three major tumor markers of PC.^37^, ^38^, ^39^, ^40^ At PAC, CA125, and CEA were risk factors for OS, and only CA125 was a risk factor for PFS (Table 2). Then, we sought to clarify the roles of the tumor marker changes. For advanced or metastatic PC, the change in CA199 has been found to be relevant to the survival prognosis.^41^, ^42^ However, no similar studies have been published at the postoperative timing. We found that a decrease in CEA indicated better OS and PFS throughout the course. We also found that among all patients, CEA, to our astonishment, kept increasing along with the processing of chemotherapy macroscopically, which further promoted the value of CEA in the prognosis prediction.

Certainly some limitations of this study were worth emphasizing. First, among the total of around 2000 postoperative PDAC patients in our center, 401 eligible patients were screened out. The incomplete follow‐up and insufficient data records were the main causes. And there could be bias accompanied with the selection. For instance, the compliance of the patients might influence the record completeness and the survival prognosis both. Second, although FBG was discovered a reliable predictor for CHC, it seemed not that satisfying to predict the CHC with only FBG according to the ROC curve. More variables and an integrated panel should be studies and proposed in the future. Third, limited by sample size and retrospective nature, the conclusion required further large‐sample multicenter data for verification.

Overall, in our study, PAC sarcopenia was found to be a risk factor for OS. Early chemotherapy administration (<34 days) was not helpful for patients with PDAC. To evaluate chemotherapy tolerance or predict CHC, a novel perspective was discovered based on PAC FBG. To monitor disease progression, CEA might be a more reliable and valuable tumor marker than CA199 and CA125. The study discussed the survival prognosis and chemotherapy tolerance at the unexplored PAC timing, which could help evaluate the PDAC patient chemotherapy strategy and predict their prognosis.

## CONFLICT OF INTEREST

The authors report no conflict of interest.

## AUTHOR CONTRIBUTIONS

*Study concepts* Ningzhen Fu and Yu Jiang; *Study design* Ningzhen Fu, Jingfeng Li, Kai Qin, and Baiyong Shen; *Data acquisition* Ningzhen Fu and Jingfeng Li; *Quality control of data and algorithms* Ningzhen Fu and Xiaxing Deng; *Data analysis and interpretation* Ningzhen Fu and Jiabin Jin; *Statistical analysis* Ningzhen Fu and Yu Jiang; *Manuscript preparation* Ningzhen Fu and Xiaxing Deng; *Manuscript editing* Ningzhen Fu and Yu Jiang; *Manuscript review* Xiaxing Deng and Baiyong Shen.

## ETHICS STATEMENT

This study was exempt from institutional review board approval due to the nature of the study. Because all data were de‐identified, patient consent was waived.

## Supporting information


FigureS1

FigureS2

TableS1

TableS2

TableS3

## Data Availability

The data where our results derived from Pancreatic Disease Center, Shanghai Jiao Tong University School of Medicine Affiliated Ruijin Hospital. The original data were not publicly available and could only be shared with the permission of the ethics committee of Ruijin Hospital.
