# Supplementary figures and images for: Who could complete and benefit from the adjuvant chemotherapy regarding pancreatic ductal adenocarcinoma? A multivariate‐adjusted analysis at the pre‐adjuvant chemotherapy timing
Source: Cancer Med. 2022 Apr 17;11(18):3397–406. doi: 10.1002/cam4.4698 (PMC9487870; doi:10.1002/cam4.4698)

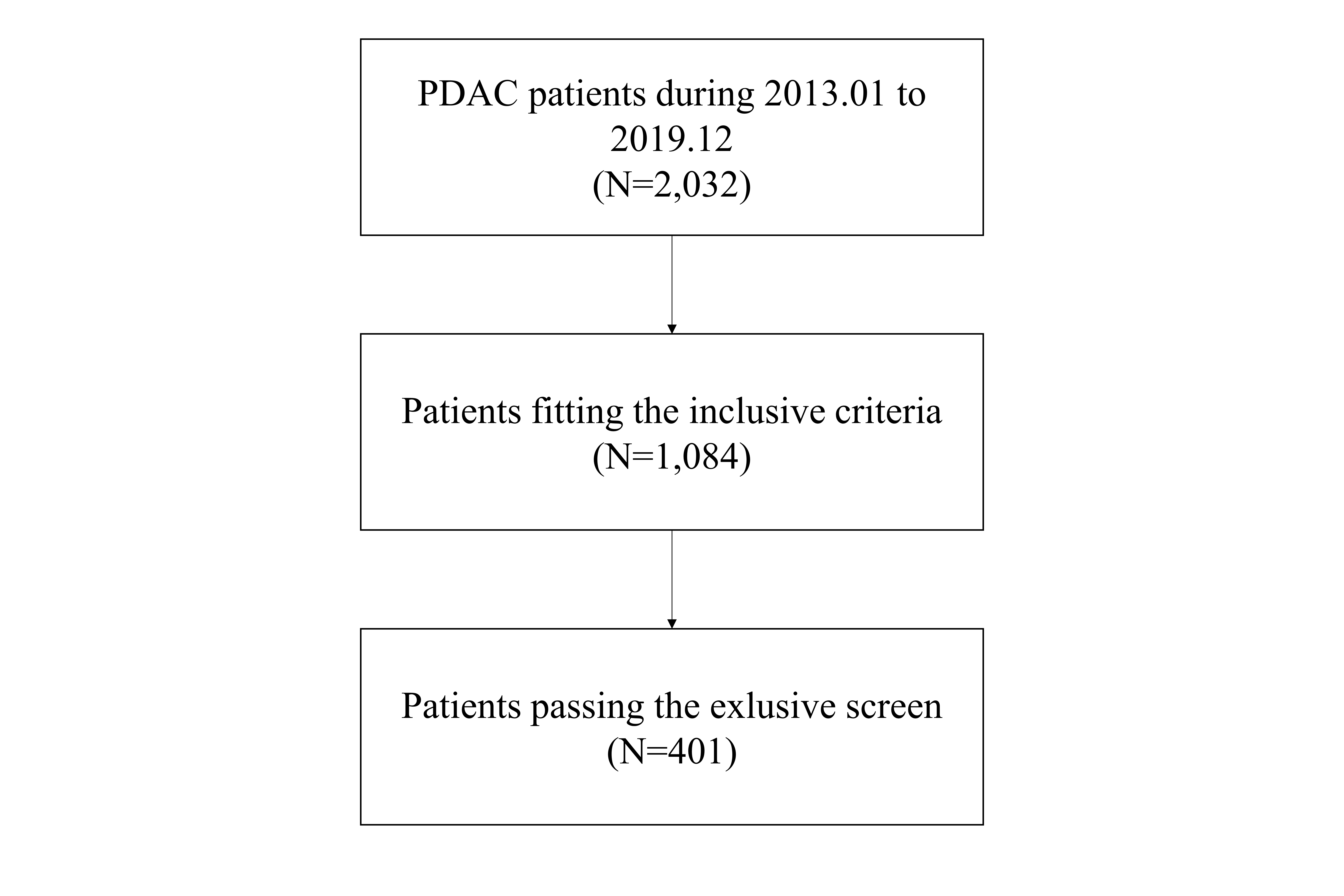

Supplement: Supplementary file 1 — FigureS1 [file CAM4-11-3397-s001.tif]

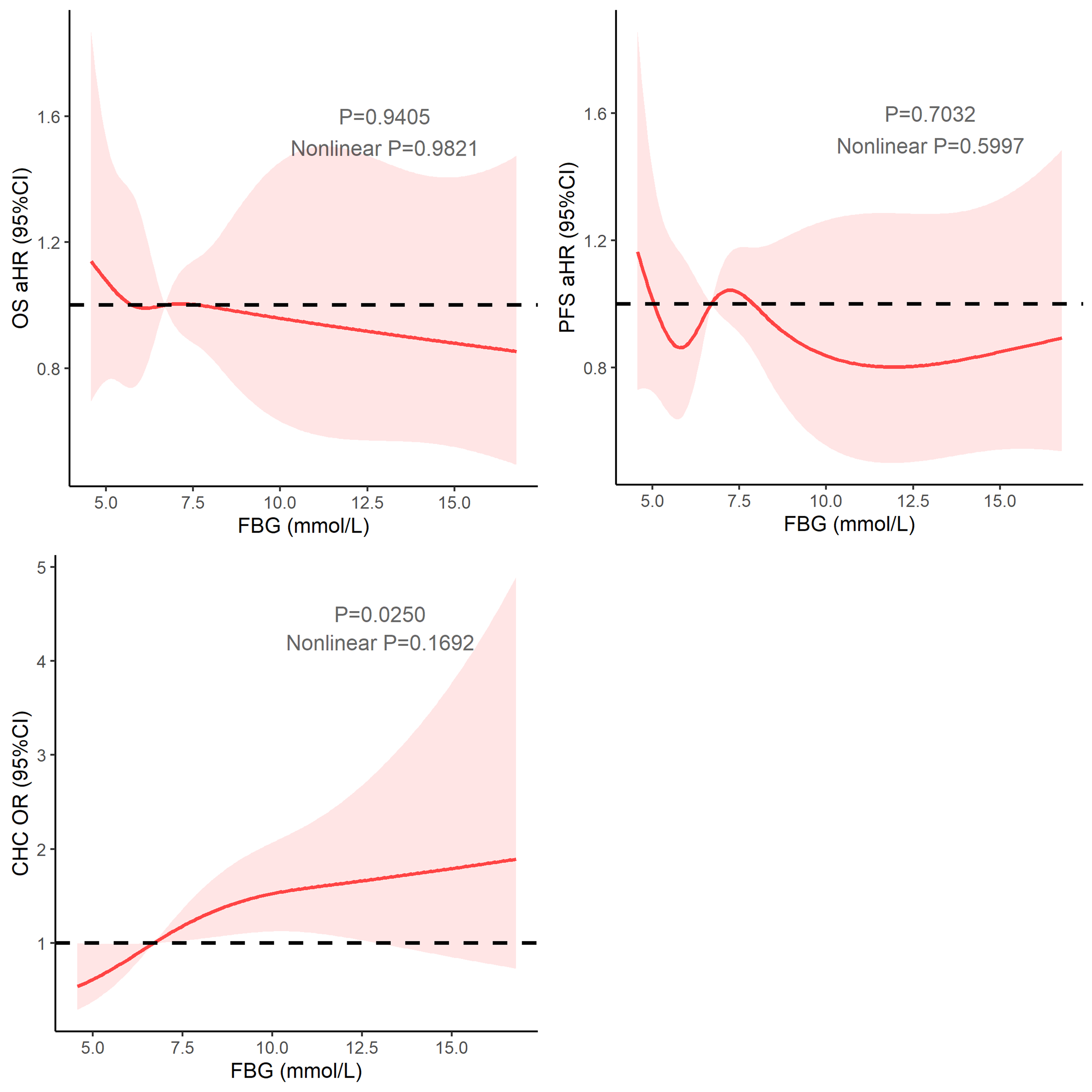

Supplement: Supplementary file 2 — FigureS2 [file CAM4-11-3397-s004.tif]
