# Supplementary material for: Who could complete and benefit from the adjuvant chemotherapy regarding pancreatic ductal adenocarcinoma? A multivariate‐adjusted analysis at the pre‐adjuvant chemotherapy timing
Source: Cancer Med. 2022 Apr 17;11(18):3397–406. doi: 10.1002/cam4.4698 (PMC9487870; doi:10.1002/cam4.4698)
Supplement: Supplementary file 3 — TableS1 [file CAM4-11-3397-s002.docx]

Supp. Table 3. The reasons and outcomes of why patients quit the initial regimen.

|  | CHC | | P value |
| --- | --- | --- | --- |
| Reasons | LH | MH | 0.226 |
| Recurrence | 30 | 25 |  |
| Intolerance | 97 | 122 |  |
| Outcomes |  |  | 0.743 |
| Quit chemotherapy | 91 | 109 |  |
| Change regimens | 36 | 38 |  |
| CHC, chemotherapy completeness; LH, less-than-half; MH, more-than-half. | | | |
