# Supplementary material for: Who could complete and benefit from the adjuvant chemotherapy regarding pancreatic ductal adenocarcinoma? A multivariate‐adjusted analysis at the pre‐adjuvant chemotherapy timing
Source: Cancer Med. 2022 Apr 17;11(18):3397–406. doi: 10.1002/cam4.4698 (PMC9487870; doi:10.1002/cam4.4698)
Supplement: Supplementary file 4 — TableS2 [file CAM4-11-3397-s003.docx]

Supp. Table 1. Kolmogorov-Smirnov normality analysis for PAC variables.

|  | BW | BMI | CA199 | CA125 | CEA | PreAlb | Alb | FBG |
| --- | --- | --- | --- | --- | --- | --- | --- | --- |
| P value | 0.007 | 0.200 | <0.001 | <0.001 | <0.001 | 0.008 | <0.001 | <0.001 |
| PAC, pre adjuvant chemotherapy; BW, body weight; BMI, body mass index; PreAlb, prealbumin; Alb, albumin; FBG, fasting blood glucose. | | | | | | | | |
