# Supplementary material for: Who could complete and benefit from the adjuvant chemotherapy regarding pancreatic ductal adenocarcinoma? A multivariate‐adjusted analysis at the pre‐adjuvant chemotherapy timing
Source: Cancer Med. 2022 Apr 17;11(18):3397–406. doi: 10.1002/cam4.4698 (PMC9487870; doi:10.1002/cam4.4698)
Supplement: Supplementary file 5 — TableS3 [file CAM4-11-3397-s005.docx]

Supp. Table 2. Wilcoxon Rank Sum Analyses of continuous variable changes.

|  | CA199 | | CA125 | | CEA | | BMI | | BW | | Alb | | PreAlb | | FBG | |
| --- | --- | --- | --- | --- | --- | --- | --- | --- | --- | --- | --- | --- | --- | --- | --- | --- |
|  | First 3 months | Last 3 months | First 3 months | Last 3 months | First 3 months | Last 3 months | First 3 months | Last 3 months | First 3 months | Last 3 months | First 3 months | Last 3 months | First 3 months | Last 3 months | First 3 months | Last 3 months |
| Average Rank Sum | -4.5 | 1.48 | -20.9 | -15.78 | 56.36 | 17.21 | -31.21 | 8.76 | -25.79 | 2.14 | -33.83 | -0.7 | 3.13 | 2.62 | 0.27 | 0.82 |
| P value | 0.008 | 0.001 | <0.001 | 0.001 | <0.001 | 0.002 | <0.001 | 0.097 | <0.001 | 0.097 | <0.001 | 0.211 | 0.758 | 0.216 | 0.55 | 0.626 |
| BMI, body mass index; BW, body weight; Alb, albumin; PreAlb, prealbumin; FBG, fasting blood glucose. | | | | | | | | | | | | | | | | |
